# Genetic and morphological divergence at a biogeographic break in the beach-dwelling brooder *Excirolana hirsuticauda* Menzies (Crustacea, Peracarida)

**DOI:** 10.1186/s12862-019-1442-z

**Published:** 2019-06-11

**Authors:** Pilar A. Haye, Nicolás I. Segovia, Andrea I. Varela, Rodrigo Rojas, Marcelo M. Rivadeneira, Martin Thiel

**Affiliations:** 1 Departamento de Biología Marina, Facultad de Ciencias del Mar, Universidad Católica del Norte, Larrondo, 1281 Coquimbo, Chile; 2 Núcleo Milenio de Ecología y Manejo Sustentable de Islas Oceánicas (ESMOI), Universidad Católica del Norte, Coquimbo, Chile; 3 Centro de Estudios Avanzados en Zonas Áridas (CEAZA), Coquimbo, Chile

**Keywords:** Southeast Pacific, Chile, Phylogeography, Population genetics, Speciation, Genital characters, Morphological variation

## Abstract

**Background:**

There is a biogeographic break located at 30°S in the southeast Pacific, in a coastal area of strong environmental discontinuities. Several marine benthic taxa with restricted dispersal have a coincident phylogeographic break at 30°S, indicating that genetic structure is moulded by life history traits that limit gene flow and thereby promote divergence and speciation. In order to evaluate intraspecific divergence at this biogeographic break, we investigated the genetic and morphological variation of the directly developing beach isopod *Excirolana hirsuticauda* along 1900 km of the southeast Pacific coast, across 30°S.

**Results:**

The *COI* sequences and microsatellite data both identified a strong discontinuity between populations of *E. hirsuticauda* to the north and south of 30°S, and a second weaker phylogeographic break at approximately 35°S. The three genetic groups were evidenced by different past demographic and genetic diversity signatures, and were also clearly distinguished with microsatellite data clustering. The *COI* sequences established that the genetic divergence of *E. hirsuticauda* at 30°S started earlier than divergence at 35°. Additionally, the three groups have different past demographic signatures, with probable demographic expansion occurring earlier in the southern group (south of 35°S), associated with Pleistocene interglacial periods. Interestingly, body length, multivariate morphometric analyses, and the morphology of a fertilization-related morphological character in males, the *appendix masculina*, reinforced the three genetic groups detected with genetic data.

**Conclusions:**

The degree of divergence of *COI* sequences, microsatellite data, and morphology was concordant and showed two geographic areas in which divergence was promoted at differing historical periods. Variation in the *appendix masculina* of males has probably promoted reproductive isolation. This variation together with gene flow restrictions promoted by life history traits, small body size, oceanographic discontinuities and sandy-beach habitat continuity, likely influenced species divergence at 30°S in the southeast Pacific coast. The degree of genetic and morphological differentiation of populations to the north and south of 30°S suggests that *E. hirsuticauda* harbours intraspecific divergence consistent with reproductive isolation and an advanced stage of speciation. The speciation process within *E. hirsuticauda* has been shaped by both restrictions to gene flow and a prezygotic reproductive barrier.

**Electronic supplementary material:**

The online version of this article (10.1186/s12862-019-1442-z) contains supplementary material, which is available to authorized users.

## Background

Biogeographic breaks are areas of shifts in species composition and represent the limits of biogeographic regions. These discontinuities are often located in areas with prominent topographical features or steep environmental discontinuities. Along the southeast Pacific coast, biogeography of marine communities is characterized by two major biogeographic breaks. The southern break is located at 42°S and corresponds to the northern limit of the Magellan Province that is dominated by sub-Antarctic species and is characterized by a topography of fjords, islands and channels, with strong influence of rivers and glaciers [1]. Also occurring at 42°S is the bifurcation of the West Wind Drift Current into the Humboldt Current System (HCS) to the north, and the Cape Horn Current towards the south. Additionally, the northern limit of the ice sheet during the last glacial period was located at 42°S.

To the north of 42°S the coast is mainly linear and under the influence of the HCS [1, 2]. The second biogeographic break is along the linear portion of the coast, at 30°S. At this latitude, there are no physical barriers or divergence of currents that may explain the presence of a biogeographic break. The 30°S biogeographic break divides to the north, the Peruvian Province, and to the south, the Intermediate Area (between 30°-42°S), and represents the limits of the geographic range of distribution of temperate and warm-affinity taxa, respectively [1, 2].

The HCS is an eastern boundary system with coastal wind-driven upwelling [3]. Wind, upwelling, and offshore transport of upwelled waters are enhanced at 30°S, which corresponds to the average location of the subtropical anticyclone [2, 4, 5]. These features create extensive changes in kinetic energy of surface waters, temperature, salinity, oxygen concentration, and in thermal seasonal variations [6, 7]. Associated with the discontinuities in environmental characteristics at 30°S, there are important changes in the structure of marine communities and in larval recruitment patterns, including a significantly lower larval recruitment and adult abundance [8–12].

A common pattern along biogeographic breaks is a concordant genetic discontinuity, or phylogeographic break, of intraspecific lineages [13, 14]. Phylogeographic breaks result from ancient and sometimes ongoing barriers to gene flow that promote divergence [15]. Concordance in the location of intraspecific genetic discontinuities suggest that there are common and persistent factors that promote intraspecific genetic structure [16], particularly for species with low dispersal capability [17–24]. Even though many factors influence intraspecific patterns of genetic diversity, including historical factors, developmental mode, planktonic larval duration, habitat availability and environmental characteristics, the intrinsic dispersal potential of a species often has the strongest influence on intraspecific genetic structure. The fact that dispersal potential is so relevant emphasizes the role of barriers to gene flow at biogeographic breaks as promoters of intraspecific divergence and speciation [20, 24–27].

In concordance with patterns detected for other marine biogeographic breaks, the 30°S break in the southeast Pacific also harbours phylogeographic breaks in species with low dispersal potential, including algae [28], intertidal barnacles [29, 30], gastropods [24, 31], and the amphipod *Orchestoidea tuberculata* [24]. All of these species have limited dispersal potential, either because they have short-lived spores such as algae, or relatively short-lived dispersive larval stages, or completely lack dispersive stages, as is the case of *O. tuberculata*. Concordant intraspecific phylogeographic breaks in species with low dispersal potential suggest that the 30°S biogeographic break is a barrier to gene flow that originated in the past [20].

Strong environmental discontinuities, as those encountered at some biogeographic breaks, promote speciation because they maintain isolation of populations to each side of the biogeographic break [32]. In the context of marine speciation, phylogeographic breaks can be considered as a stage of the speciation process, which can be understood as a continuum between a panmictic population and the complete reproductive isolation between two or more populations [33, 34]. In a scenario where gene flow restrictions are persistent in time, genetic drift will lead to increasing intraspecific divergence over time on either side of the barrier [24, 35]. Along the speciation divergence gradient, advanced stages are characterized by strong reproductive isolation, genetic discontinuities and lineage sorting with reciprocal monophyly [36]. Intraspecific divergence and speciation are more likely to occur with more extreme environmental discontinuities, with greater temporal persistence of physical discontinuities, and/or with lower dispersal potential [36]. Estimates of intraspecific divergence of marine benthic taxa at the 30°S biogeographic break indicate that it may have started as early as the Pleistocene, over 200,000 years ago [24, 37]. Taxa that have larvae that spend less than two weeks in the water column also display divergence and the onset has been dated during or soon after the last glacial period [24]. The 30°S phylogeographic break is deeper in taxa with very low dispersal potential suggesting that for those taxa the break’s onset was thousands of years ago. The ancient onset of the break and the current environmental discontinuities that occur at 30°S, make this phylo- and bio-geographic break a natural scenario for the evaluation of intraspecific divergence processes.

*Excirolana hirsuticauda* Menzies (Crustacea, Peracarida) is a beach-dwelling isopod of > 12 mm of body length with low dispersal potential and a wide geographic distribution (ca. 22°S-43°S), crossing the 30°S biogeographic break. Shore-dwelling peracarids generally display strong spatial genetic structure [38–42] and phylogeographic discontinuities coincident with biogeographic breaks [23]. The sympatric congener of *E. hirsuticauda*, *E. braziliensis,* harbours three reciprocally monophyletic lineages, two of which are parapatric at ~ 30°S [43, 44].

In this study, we focus on the 30°S latitude in the southeast Pacific as a natural laboratory to explore intraspecific lineage divergence in *E. hirsuticauda* at a biogeographic break. We analysed genetic variation of mitochondrial DNA sequences and microsatellite loci, and morphological and morphometric variation along most of the distributional range of this species (25°S - 42°S). Previous studies in marine peracarids that incorporate morphological analyses in addition to the genetic counterpart suggest that morphology usually correlates with genetic divergence [45–47]. Using genetic and morphological divergence, herein we test the hypothesis that *E. hirsuticauda*, a species with limited dispersal, has a phylogeographic break coincident with the 30°S biogeographic break, and that the degree of genetic and morphologic divergence represents an advanced stage of speciation at 30°S.

## Results

### Mitochondrial DNA

**Fig. 1:**
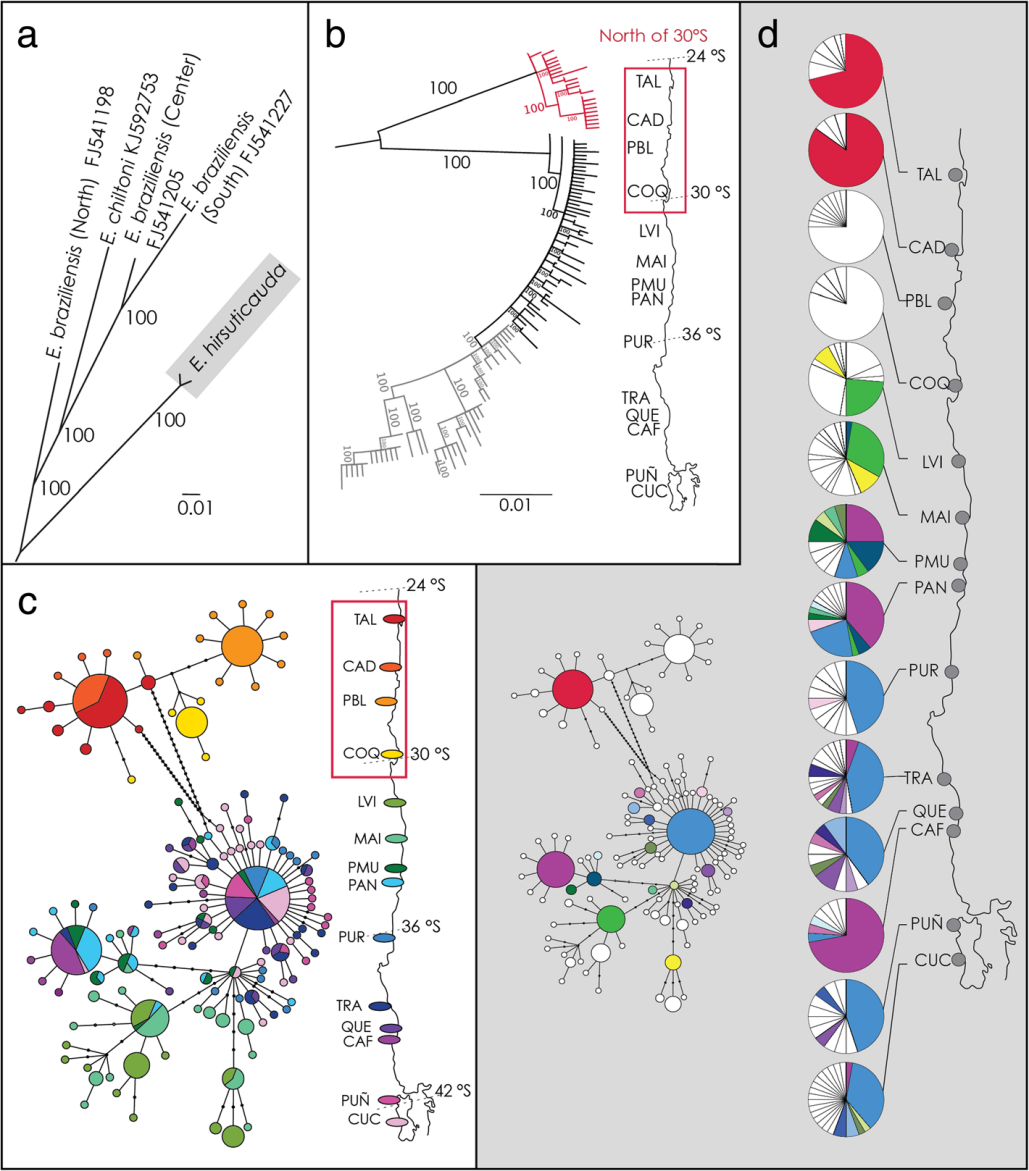
Genetic structure based on *COI* haplotypes of 404 individuals of 14 populations of *E. hirsuticauda*. (**a**) Maximum likelihood phylogram of the relationships between some *Excirolana* species based on *COI* sequences. Sequences for *E. braziliensis* and *E. chiltoni* were obtained from GenBank (accession numbers at tip labels). (**b**) Maximum likelihood phylogram of *COI* haplotypes of *E. hirsuticauda*. Clade in red was unique to the sites to the north of 30°S (marked with red square on map) and showed reciprocal monophyly with sites to the south of 30°S. Grey branches and labels indicate a derived linage, albeit without reciprocal monophyly. In (**a**) and (**b**) numbers along the branches represent bootstrap support values. (**c**) Haplotype network of *COI* sequences. Circles represent each haplotype with size proportional to each colour representing a locality as designated in the contiguous map. (**d**) Geographic distribution of haplotypes. Each pie represents a haplotype, and the subdivisions represent relative frequencies of each haplotype per site. Colours represent shared haplotypes according to haplotype network in grey inset box. White portions denote haplotypes not shared with other localities

The analysed *Cytochrome Oxidase I* (*COI)* sequence dataset was composed of 404 sequences of 600 base pairs of length from 14 localities, which corresponded to 128 distinct haplotypes (Table 1, Additional file 1). The inferred best model of molecular evolution for *COI* was the GTR + G + I model. Phylogenetic reconstructions supported the monophyly of *E. hirsuticauda* (Fig. 1a) and revealed two reciprocally monophyletic intraspecific lineages; one to the north and one to the south of 30°S (Fig. 1b). A third group of haplotypes, albeit not reciprocally monophyletic, was detected embedded within the clade located to the south of 30°S (Fig. 1b). The haplotype network revealed the same two main clades, separated by at least 24 mutational steps (Fig. 1c). To the south of 30°S there was the third group of divergent haplotypes occurring mainly in the central study area, between 30°S and 35°S (Fig. 1c, d). Given that most analyses were performed considering these groups, we will refer to them as: North group (north of 30°S), Center group (between 30 and 35°S) and the South group (to the south of 35°S).

**Table 1:** Summary of samples of *Excirolana hirsuticauda* used for *COI* analyses, and genetic diversity and neutrality tests for *COI* sequences of *Excirolana hirsuticauda*

				Diversity indices					Neutrality tests		Demo. expansion		Geo. expansion	
		Latitude	N	*S*	*H*	*Rh*	π	*k*	*D*	*F*	*P* (SSD)	*P* (Hr)	*P* (SSD)	*P* (Hr)
Taltal	TAL	25°70′S	38	6	7	4.303	0.0010	0.609	**−1.548**	**−4.280**	0.532	0.505	0.319	0.533
Caldera	CAD	26°59′S	20	3	4	3.000	0.0005	0.300	**−1.723**	**−2.749**	0.455	0.595	0.450	0.611
Playa Blanca	PBL	28°11′S	36	12	10	5.000	0.0011	0.667	**−2.419**	**−8.953**	0.333	0.514	0.168	0.517
Coquimbo	COQ	29°54′S	20	9	5	4.000	0.0017	0.989	**−2.098**	−1.142	0.198	0.450	0.569	0.655
Los Vilos	LVI	31°51′S	38	21	11	6.841	0.0078	4.700	−0.200	0.172	0.449	0.614	0.573	0.856
Maitencillo	MAI	32°38′S	39	27	16	9.848	0.0087	5.225	−0.622	−2.640	0.009	0.001	0.060	0.081
Pichilemu	PMU	34°23′S	20	16	12	11.000	0.0071	4.242	−0.221	−3.411	0.291	0.418	0.192	0.658
Pangua	PAN	34°29′S	36	25	14	8.174	0.0068	4.073	−1.118	−2.725	0.163	0.166	0.289	0.564
Purema	PUR	36°26′S	20	18	12	11.000	0.0036	2.142	**−2.174**	**−7.309**	0.459	0.593	0.498	0.674
Tranaquepe	TRA	38°10′S	36	26	18	10.460	0.0041	2.462	**−2.101**	**−11.785**	0.881	0.981	0.884	0.981
Queule	QUE	39°22′S	20	18	11	10.000	0.0040	2.384	**−1.994**	**−5.080**	0.572	0.862	0.610	0.861
Calfuco	CAF	39°46′S	25	14	8	5.600	0.0039	2.320	−1.299	−1.159	0.001	0.999	0.713	0.782
Puñihuil	PUÑ	41°55′S	20	17	12	11.000	0.0028	1.700	**−2.414**	**−8.927**	0.968	0.894	0.976	0.890
Cucao	CUC	42°40′S	36	25	22	12.175	0.0039	2.333	**−2.113**	**−20.55**	0.360	0.554	0.292	0.575
Total			404	114	128	13.757	0.0041	2.439						

Genetic diversity measures for 14 local populations used for *COI* sequences analysis: Samples size (N), number of segregating sites (*S*), number of haplotypes (*H*), haplotype richness after rarefaction (*Rh*), nucleotide diversity (π), average number of nucleotide differences between pairs of sequences (*k*). Demographic inference analyses: neutrality tests [Tajima’s *D* (*D*) and Fu & Li’s *F* (*F*)] (significant values are in bold), probability values associated with Sum of Squares Deviation of the mismatch frequency distribution of the number of pairwise nucleotide differences [*P* (SSD)] and probabilities of Harpending’s Raggedness index [*P* (Hr)]. Both SDD and Hr were calculated based on expected mismatch distribution according to the demographic (Demo. expansion) and geographic expansion (Geo. expansion) models

Most population pairwise Φ_*ST*_ values were high and significant (Additional file 2). *Analysis of Molecular Variance* (AMOVA) determined that 78.06% of the genetic variance was explained by a priori defined groups (North, Center and South) (Table 2). Migration rates from IMa2 analyses revealed that the North and Center groups were strongly isolated and started diverging ~ 1.4 million years ago. The Center and South groups started diverging more recently, ~ 72,000 years ago (Table 3, Additional file 3). No migration was detected between North and Center groups and very low and asymmetrical migration between Center and South groups, with a slight southern direction, but not statistically different from the null model of no migration (Table 3, Additional file 3).

**Table 2:** AMOVA analysis for *COI* sequences of 14 local populations of *Excirolana hirsuticauda*

	DF	SS	Var. Comp.	% of Var.	Stats.
Among groups	2	2177.93	7.93	78.06	Φ_CT_ = 0.78
Among pops. Within groups	11	304.02	0.94	9.21	Φ_SC_ = 0.42
Within pops	390	503.89	1.29	12.73	Φ_ST_ = 0.87

The three groups considered were the North, Center, and South groups. All analyses yielded *P* values lower than 0.001. *DF* degrees of freedom, *SS* sum of squares, *Var. Comp* variance component, *% of Var* percentage of variance explained, *Stats* statistics

**Table 3:** Estimates of onset of splitting time (t) across the two detected genetic discontinuities (30° and 35°S) and migration rates (m) from North to South (N > S) and S to N (S > N) for *COI* sequences of *Excirolana hirsuticauda* based on the isolation-with-migration model implemented in IMa2

Model	Value	m_N > S_	m_S > N_	t (years)
North-Center	HiPt	0.0005	0.0005	1,365,000
	Mean	0.0428	0.01815	1,338,333
	95%HPD	0.1205	0.0515	4,165,000
	LRT	0.000^a^	0.000^a^	
Center-South	HiPt	0.205	0.005	72,083
	Mean	0.299	0.0532	73,925
	95%HPD	0.675	0.175	98,750
	LRT	2.178^a^	0.000^a^	

For each model and parameter, the high point (HP), mean, 95% highest posterior density (95% HPD) of the marginal posterior probabilities are shown, as well as the Likelihood Ratio Test (LRT) performed on migration rates. ^a^non-significant values

Genetic diversity differed between groups. The North group had low haplotype richness, low nucleotide diversity, and a low number of shared haplotypes. The Center group had high haplotype richness, high nucleotide diversity, and numerous shared haplotypes, while the South group had high haplotype richness, low nucleotide diversity, and low number of shared haplotypes (Table 1, Fig. 1d). Likewise, indicators of departures of DNA variability from neutrality processes expectations of the three groups differed. Tajima’s *D* and Fu & Li’s *F* (Table 1), were mostly significant in the North and South groups and not-significant in the Center group. Tajima’s *D* and Fu & Li’s *F*, calculated per group including all sites, showed that the North and South groups had highly significant values while the Center clade was not-significant (Table 4). Mismatch frequency distributions of the number of differences between pairs of sequences had higher significance in the North and South groups (Table 1). Temporal reconstructions of effective population depicted as Bayesian Skyline plots, inferred using HKY + I and TN93+ G models respectively, were similar in shape and indicative of a past population expansion for the North and South groups (Additional file 4). Altogether data show that the North and South groups underwent a past demographic expansion event. Based on a τ = 4.316 (Table 4), the estimated time since the last population expansion of the North group was 240,000 years ago, while the South group had an earlier expansion, dating back to 370,000 years ago based on τ = 6.711 (Table 4). These estimations were in agreement with the median of the estimated date for the Most Recent Common Ancestor (tMRCA) calculations carried out in BEAST. Estimations located the tMRCA of the North group at 240,000 years ago and of the South group at 340,000 years ago approximately (Additional file 4).

**Table 4:** Demographic inference analyses for *COI* sequences of 14 local populations of *Excirolana hirsuticauda*

	Neutrality Tests		Demographic Expansion		
	*D*	*F*	*P* (SSD)	*P* (Hr)	τ
North	**−1.403**	**−12.913**	0.120	0.099	4.316
Center	−1.148	**− 17.989**	0.180	0.217	7.961
South	**−2.243**	**−26.169**	0.625	0.848	6.711

Neutrality tests [Tajima’s *D* (*D*), Fu & Li’s *F* (*F*)] (significant values are in bold), probability values associated with Sum of Squares Deviation of the mismatch frequency distribution of the number of pairwise nucleotide differences [*P* (SSD)] and probabilities of Harpending’s Raggedness index [*P* (Hr)], and Tau (τ) statistic, under the demographic expansion model

### Microsatellite loci

Eight loci were genotyped for 317 individuals of eight localities (Additional file 5), however, three loci (*Ehir1*, *Ehir6* and *Ehir49*) were excluded from further analyses after corroborating that they were in significant linkage disequilibrium and deviated from Hardy-Weinberg Equilibrium (HWE) across all populations (data not shown).

The Brookfield method in MICRO-CHECKER found no scoring errors or linkage disequilibrium for the five remaining loci (Additional file 6). HWE analyses using the 5 remaining loci at the population level indicated that most localities were out of HWE. Inspection by locus and population shows that loci had less than 50% of localities out of HWE (Additional file 7). Loci were highly polymorphic, with a total Polymorphic Information Content (PIC) of 0.810, and with similar observed and expected heterozygosities, ranging from 0.438 to 0.855 and 0.467 to 0.923, respectively (Additional file 8). Overall, locus-by-locus analyses showed non-significant *F*_*IS*_ values, while population-by-population analyses showed significant *F*_*IS*_ values, indicating population processes may be determining the heterozygosities (Additional file 9).

**Fig. 2:**
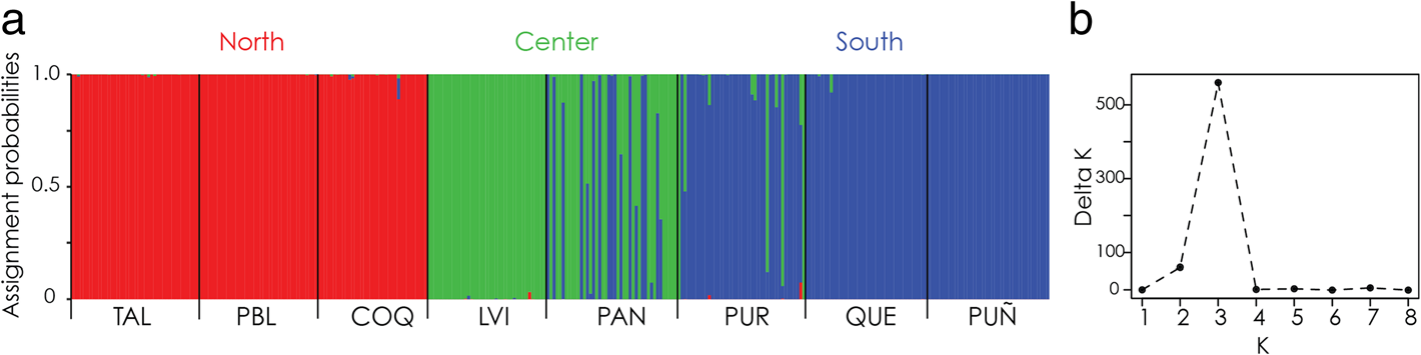
Bayesian assignment of individuals of *E. hirsuticauda* to three detected genetic groups based on microsatellite data. (**a**) Assignment of each individual to genetic clusters is represented with 3 colours. (**b**) Best-*k*, optimal number of clusters, according to the estimation based on Evanno’s (Evanno et al. 2005) method

Individual-based Bayesian clustering analysis without a priori information of origin location, performed in STRUCTURE, grouped genotypes in three population clusters (Fig. 2) that corresponded to the three groups detected with *COI* sequences (North of 30°S, Center between 30 and 35°S, and South of 35°S) (Fig. 2). Population pairwise *F*_*ST*_ values were mostly significant (Additional file 10). AMOVA for three groups (the same ones tested for *COI* data) revealed that most of the molecular variance of microsatellite loci was explained by groups (79.39%) (Table 5).

**Table 5:** AMOVA analysis for microsatellite loci of eight local populations of *Excirolana hirsuticauda*

	DF	SS	Var. Comp.	% of Var.	Stats.
Among groups	2	954.87	1.79	79.39	Φ_CT_ = 0.14
Among pops. Within groups	5	61.89	0.16	7.03	Φ_SC_ = 0.08
Within populations	225	133.84	0.31	13.58	Φ_ST_ = 0.21

The three groups considered were the North, Center, and South groups. All analyses yielded *P* values lower than 0.001. *DF* degrees of freedom, *SS* sum of squares, *Var. Comp*. variance component, *% of Var* percentage of variance explained, *Stats.* statistics

### Morphology and morphometrics

**Fig. 3:**
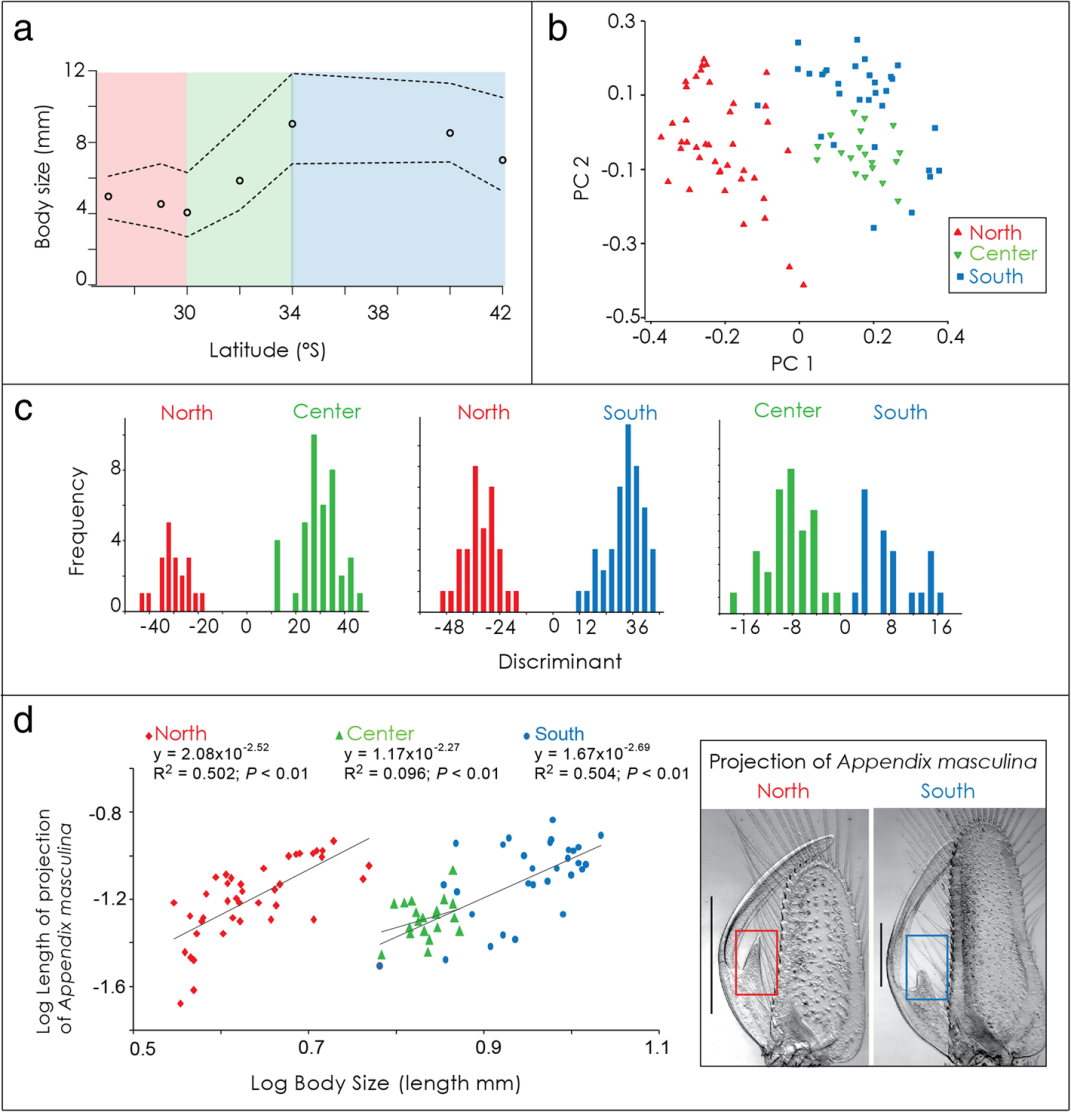
Body size distribution and morphometric differentiation analyses of *Excirolana hirsuticauda*. (**a**) Latitudinal variation in total body length, showing the mean (circles) and 95th and 5th percentiles (broken lines). Colours in the graph represent the three groups of populations detected with genetic analyses (North, Center and South). (**b**) Plot of principal component analysis for 90 males. Symbols indicate from which group individuals were collected. (**c**) Results of Hotelling’s discriminant test (T^2^) corroborating the ordination of PCA analysis. (**d**) ANCOVA results showing the relationship between body size and length of projection of *appendix masculina* for each group of populations. Insert pictures correspond to detail of the morphology of the *appendix masculina* of males from North and South groups. Scale bars represent 0.5 mm

A total of 3362 isopods were measured from seven sites (Additional file 11). Total body length ranged from 2.5 mm to 13.2 mm (Fig. 3a, Additional file 12). Individuals of the North group were smaller than individuals belonging to the Center and South groups (*P* <  0.01). Total body length was significantly different both between the three groups, with groups explaining 61% of variation in total body length, and between sites within groups, although sites explained only 12% of the variation (Nested ANOVA, F: 211.2; df: 4 74.36; *P* <  0.01) (Table 6).

**Table 6:** Summary of nested ANOVA on total body length of 3362 individuals of *Excirolana hirsuticauda*

Factor	DF	SS	MS	F	*P*	%Var
Group	3	223.09	74.36	2182.4	< 0.01	61
Group:Site	4	28.78	7.20	211.2	< 0.01	12

*DF* degrees of freedom, *SS* sum of squares, *MS* mean of squares, *%Var* % of variance explained. Group represents the three groups (regions) as fixed variable, and Group:Site, corresponds to sites nested within regions, with Site as random variable

Principal component analysis (PCA) of adjusted morphometric data of males revealed a sharp difference between the North, Center and South groups (Fig. 3b) that was significant according to Hotelling’s discriminant test (*P* <  0.01) (Fig. 3c). The first principal component (PC) explained 55.1% of the variation and combined with PC 2 they explained 77.3% of the variation. In PC 1, the character with the highest contribution was the lateral projection of the *appendix masculina* (− 0.742) (Additional file 13). The analysis was redone after eliminating the discrete variables, but the results were the same (data not shown). Permutation Analysis of Variance (PERMANOVA) performed with the three detected groups revealed significant differences between them (df = 2; Pseudo-F = 39.872; P(perm) = 0.0001). Pairwise comparisons showed the same pattern between North-Center groups (P(perm) = 0. 0001), North-South (P(perm) = 0.0001) and with a lower distance, between Center-South groups (P(perm) = 0.0023) (Table 7).

**Table 7:** PERMANOVA analysis testing morphological differences between the three detected groups of *Excirolana hirsuticauda* in the PCA analysis (see Additional file 13)

Model	Source	DF	SS	MS	Pseudo-F	*P-value*
Total Model	Groups	2	35.696	17.848	39.872	0.0001*
	Residual	87	38.943	0.045		
	Total	89	74.639			
Pairwise Comparisons	Groups				*t*	*P*-value
	N, C				68.829	0.0001*
	N, S				7.373	0.0001*
	C, S				22.552	0.0023*

Total PERMANOVA model including the three groups (North, Center, and South groups) and pairwise comparisons between groups. Table include degrees of freedom (DF), sum of squares (SS), mean of squares (MS), Pseudo-F ratio and *p-values*. *P-values* were calculated with 9999 permutations of the residual model [48]. * significant values

Analysis of Covariance (ANCOVA) on Log_10_-transformed variables evaluated on selected morphometric characters demonstrated that, accounting for body length, the lateral projection of the *appendix masculina* was the only character that was different between groups (Fig. 3d); other slopes showed no differences between groups. Individuals belonging to the northern group showed relatively larger projections than individuals from the southern groups, despite their smaller body length (ANCOVA on Log_10_-transformed data, F: 62.28; df: 1; P <  0.01) (Table 8).

**Table 8:** Results from ANCOVA on Log10 transformed variables evaluated on four selected morphometric characters of *Excirolana hirsuticauda*

Relationship	Intercept			
	*F*	DF	MS	*P*
TBL / Length of the lateral projection of AM	62.28	1	0.99	< 0.01
TBL/ AM length	3.098	1	0.02	> 0.05
TBL/ Interocular distance	2.75	1	0.004	> 0.05
TBL/ Length of peduncle A2	10.37	1	0.007	< 0.01

Table includes F-ratio (*F*), degrees of freedom (DF); mean of squares (MS); total body length (TBL); *appendix masculina* (AM)

## Discussion

### Divergence of *Excirolana hirsuticauda*

Phylogeographic and morphological analyses of the beach-dwelling isopod *E. hirsuticauda* along the southeast Pacific coast showed concordant genetic and morphological divergence at 30°S and at 35°S. The strongest divergence, both with genetic and morphological markers, was detected across the 30°S biogeographic break, where the degree of divergence is congruent with a very advanced stage of the speciation process, that started over a million years ago. The significant differentiation of a reproductive character suggests that there may be reproductive isolation and complete speciation to the north and south of 30°S.

Intraspecific lineage divergence at 30°S has been likely maintained by the oceanographic discontinuities at 30°S that promote ecological shifts [5, 8, 11] and divergent selection with local adaptation [49]. Another variable that may enhance divergence is the detected habitat discontinuity across the study area [50–52]. Habitat connectivity values, determined from analysis of the length and distance between sandy beaches distributed between 25°S and 42°S, showed lower habitat connectivity at ~ 30–31°S, with a value up to eight orders of magnitude lower than in the rest and with overall less and lower values (Additional file 14). Indeed, there are very few and only small sandy beaches between 30 and 31°S (see also [2]), creating an important habitat discontinuity. Habitat discontinuity has been invoked to explain phylogeographic breaks in other marine organisms such as algae [53–55], which have very low dispersal potential, and also for the isopod *Jaera albifrons* (38). Further studies that directly evaluate habitat connectivity along the HCS will allow determining the contribution of habitat continuity in the phylogeographic structure of marine taxa at 30°S. The major constrains to gene flow detected herein are likely a combination of low habitat connectivity, oceanographic discontinuities [2–12], the very limited dispersal potential and small body size of *E. hirsuticauda*.

A second discontinuity in population divergence of *E. hirsuticauda* was detected between 34°23′S and 36°26′S (~ 35°S). Intraspecific divergence at 35°S was weaker than at 30°S, and started more recently (less than 100,000 years ago). At the 30°S biogeographic break, genetic data detected reciprocally monophyletic groups to the North and South, while at 35°S the divergence did not lead to reciprocal monophyly. In spite of the weaker overall signal, the 35°S break was evident with both genetic and morphological data.

Although the presence of a genetic discontinuity at 35°S in a marine species of the coast of Chile is novel, it is in keeping with species boundaries known to occur at this location. The closest described genetic discontinuity is between 32°S and 34°S for the seaweed *Mazzaella laminarioides* [28]. Based on the distribution of almost 1000 species, Lancellotti & Vásquez [56, 57] described a discontinuity in species distribution where the northern limit of a transition zone was at 35°S. This transition zone extends south to 41°S and is characterized by gradual species replacement and changing environmental conditions. In particular, short-ranged, warm-temperate species have their southern limit of distribution at ~ 35°S [58]. Analysing peracarid diversity, Rivadeneira et al. [59] found that species diversity decreases to the north of 35°S. The direct effects of El Niño Southern Oscillation (ENSO), as well as the warm Peruvian Countercurrent that reaches from the south up to 35°S [58], have been invoked to explain species diversity changes at 35°S [57, 59]. Recently, Lara et al. [60] reported a biogeographic break at 35°S for marine rocky intertidal species of the study area, particularly species with planktotrophic larvae. They found the break to be consistent with the spatial structure of sea surface temperature, chlorophyll-a, and river outflow. All of the above conforms relevant evidence about the existence of a genetic discontinuity and a biogeographic break at 35°S on the southeast Pacific coast for some marine species.

### Direction of population expansion and genetic diversity of genetic groups of *E. hirsuticauda*

A past population expansion event could be suggested for the North and South, but not the Center group. The demographic expansions of the North and South groups date earlier than the divergence of the Center group from the South group. The expansions date during two Pleistocene interglacial periods, 240,000 and 240,000–370,000 years ago, for the North and South groups, respectively.

The genetic diversity and likely demographic differences between North and South groups are likely outcomes of historic signatures of isolation in glacial refugia during low sea-level glaciation periods of the Pleistocene. The regional persistence of species in refugia has been commonly described in marine species (e.g. [21, 61]). Subsequently, during interglacial periods, there may have been a northern coastal recolonization explaining the more recent population expansion detected for the North clade. Lower diversity in the North clade suggests that the direction of geographic and demographic expansions of *E. hirsuticauda* were from south to north, similar as suggested for other species expanding from southern glacial refugia [62–64]. Gene flow between Center and South groups is low but asymmetric with gene flow occurring only in the south-north direction. In addition to the effects of the direction of colonization, sufficient time has passed to allow drift within populations to enhance differentiation.

Species from sandy beaches such as cirolanid isopods may locally disappear after major changes in beach morphology and height, but some species, including *E. hirsuticauda* can also rapidly recolonize sandy beaches [65]. Environmental changes such as the ones caused by more intense effects of ENSO at lower latitudes [57, 58] may have also shaped the lower genetic diversity in the north. The effects of ENSO may have led to repeated bottlenecks during El Niño phases [66], further reducing genetic divergence in the northern area.

### Speciation within *Excirolana hirsuticauda*

The degree of genetic and morphological divergence of *E. hirsuticauda* to the north and south of 30°S, with reciprocal monophyly and lack of gene flow, suggests an advanced stage of the speciation process occurring at a biogeographic break. The morphological trait that explained most of the divergence at 30°S was the *appendix masculina* of males. Isopods have internal insemination and sperm transfer is often mediated by the *appendix masculina,* a structure of the pleopod that shows great variation between species [67]. Detected groups of *E. hirsuticauda* populations showed significant divergence in the relative length (and not the absolute length) of the projection of the *appendix masculina* in relation to the rest of the appendix, which should lead (if it has not already) to a prezygotic reproductive isolation, similar as reported for *E. braziliensis* [68] and other marine invertebrates (e.g., [69, 70]). Divergence in genital characters is commonly observed in arthropods, likely enforcing reproductive isolation and speciation [71, 72]. Herein we demonstrate that the shape of the *appendix masculina* of *E. hirsuticauda* clearly distinguishes and allows the correct assignment of individuals from the north and south of the 30°S biogeographic break. Additionally, the *appendix masculina* also allows identification of linages separated at 35°S albeit with less divergence than at 30°S. There is consistency in the degree of genetic and morphological divergence in each of the two discontinuities detected, at 30°S and 35°S.

## Conclusion

The evolutionary history of *E. hirsuticauda* led to concordant signals of divergence in genetic diversity and morphology, specifically of a trait related to reproductive isolation, at two geographic areas with differing time since onset of the divergence. This study highlights that in *E. hirsuticauda*, divergence in the *appendix masculina* of males, morphometry and genetic diversity co-vary at the same latitudes. Additionally, it exemplifies the result of divergence across biogeographic breaks that can shape species diversity by restricting gene flow. Restricted gene flow resulting from the combination of small body size, mode of development without a pelagic larval stage, oceanographic and habitat discontinuities have likely shaped the divergence processes in *E. hirsuticauda* along the Humboldt Current System. The evolution of a morphological trait that could be a reproductive barrier suggests that even in the absence of ecological or habitat constrains to gene flow, reproductive isolation would persist. Thus, both restricted gene flow and the prezygotic reproductive barrier were likely factors that promoted speciation within *E. hirsuticauda*.

## Methods

### Mitochondrial *COI* gene

Individuals of *E. hirsuticauda* were collected from each of 14 localities between 25°S and 42°S (Table 1). Isopods were washed out of bulk sand samples using 2 mm mesh bags, and were preserved in 95% ethanol and stored at − 20 °C. Genomic DNA was isolated using the QIAamp DNA kit (Qiagen Inc., Valencia, CA, USA).

We obtained partial mtDNA *COI* gene sequences using the procedures described by Varela & Haye [43] using primers HCO and LCO [73]. Inspection and alignment of chromatographs were carried on with the software GENEIOUS R8 [74].

Phylogenetic analyses were carried out using Maximum Likelihood with MODELTEST in PAUP* 4.0b10 [75]. Support for nodes was estimated with 1000 non-parametric bootstrap replicates.

A median-joining haplotype network was constructed using NETWORK 4.6 (Fluxus Technology, 2010). Number of segregating sites, number of haplotypes, nucleotide diversities, Tajima’s *D* [76] and Fu & Li’s *F* [77] were calculated using DNASP 5 [78]. Haplotype richness (*Rh*), after rarefaction to the minimum sample size of *N* = 20, was estimated using CONTRIB 1.4 [79].

To detect historical population expansions, mismatch frequency distributions of the number of nucleotide differences between pairs of sequences and Raggedness index were performed in Arlequin 3.5 [80] and Bayesian Skyline plots, to infer population dynamics through time, in BEAST v 2.4.8 [81, 82]. In BEAST, we used an uncorrelated log normal relaxed clock model for each of the detected groups with five independent runs using 100 × 10^6^ generations sampled every 1000 generations. For each group, we performed separately a JModelTest v2 [83] in order to use the best fit substitution model for the demographic reconstruction. Conversed chains were checked with Tracer v 1.6 (http://beast.community/tracer). Tracer v 1.6 was used to depict the Bayesian skyline plots. For this, we used the median values and corresponding 95% HDP (highest density probabilities) confidence intervals for effective population size (N_e_) dynamics trough time and the time of the most recent common ancestor (tMRCA) of the demographic expansion for each group.

Pairwise Φ_*ST*_ values were estimated using ARLEQUIN 3.5. ARLEQUIN 3.5 was used to perform AMOVA using a priori groups detected by previous analyses using the pairwise difference as distance method with 10,000 permutations.

To estimate the splitting time (t) of the detected genetic groups and the degree of isolation of each group (migration patterns), we used the isolation-with-migration model implemented in the software IMa2 [84, 85]. For these purposes, we carried out several preliminary runs in the M mode of the software to determinate the best set of priors that ensure mixing and convergence of the Markov Chains (MCMC). Uniform priors were used to estimate splitting time (t = 100), whereas an exponential prior (mean = 1) for gene flow (m) was adopted. We executed 1 × 10^8^ MCMC iterations sampling every 100 generations, with a burn-in period of the first 25%. According to the authors’ recommendation, we assumed that the mutation rate is under a Hasegawa-Kishino-Yano (HKY) model. After achieving convergence in the M-mode, we used the simulated genealogies using the L-Mode (Load Tree mode) to calculate the log maximum-likelihood and credibility intervals (95% under HPD) estimates for migration parameters using a Likelihood Ratio Test (LRT). Splitting times were rescaled into years (t/μ) and effective rate of migration (Θ_x_/ m_x_)/2) (rate at which genes come into population, per generation) using a mutational rate of 2% per Myr [86, 87].

### Microsatellite loci

For microsatellite DNA analyses, eight primer pairs [88] were used to assess the degree of population differentiation of *E. hirsuticauda* from eight localities. Amplicons were run on an automated sequencer and allele sizes were scored using GENEMAKER 1.80 (SoftGenetics). MICRO-CHECKER 2.2.3 [89] was used to detect scoring errors and null alleles using the Brookfield 1 method [90].

GENEPOP 4.0 [91] was used to test for linkage disequilibrium and deviations from HWE expectations within each population and per locus, followed by sequential Bonferroni correction. Allele richness estimates were obtained with a rarefaction analysis using HP-RARE v1.0 [92]. The following analyses were also performed: estimation of heterozygosities and proportion of polymorphic loci (GENALEX [93]); estimation of inbreeding coefficients *F*_*IS*_ (F_STAT_ 2.9.3.2 [94]); estimation of number of groups with SAMOVA 1.0. Finally, the Bayesian method of Prichard et al.*,* [95] implemented in STRUCTURE 2.3.4 was used to investigate the spatial genetic structure. The most likely value for *K* was assessed using Evanno’s method [96] by comparing the likelihood of the data for different values of *K*. Calculations were conducted from K 1–9 with 10 replications using an ancestry model that incorporates admixture, each one with a burn-in period of 25%, followed by 5 × 10^5^ iterations.

### Morphology and morphometrics

At least 200 individuals were collected at each of seven beaches between 26.99°S and 42.66°S. Since a recent study had confirmed that body sizes of *E. hirsuticauda* increased with latitude [97], herein we determined the body length for both males and females. For each specimen, total body length from the tip of the rostrum to the posterior end of the pleotelson was measured using a dissecting microscope with a graded ocular (Additional file 15).

In a subset of the collected males (10 per population, but 20 males at the populations just north and south of 30°S), 19 measures of body morphology (Additional file 15) were taken. Sample sizes of 10–25 adults have been determined as adequate to characterize the morphometry of populations of *E. braziliensis* Weinberg & Starczak. Especially since the appendages involved in sperm transfer might vary among populations (e.g. [72, 98]), we dissected the second pleopod and measured the length of the endopod, *appendix masculina,* and the lateral projection (i.e. “deep notch” in: Ribetti & Roccatagliata [98]). Dissected appendages were placed in a drop of glycerine on a microscope slide and covered with a cover slide before taking digital images through a microscope. Images were analysed with the program IMAGE-PRO PLUS 6.0 (Media Cybernetics, Inc.).

Nested ANOVA was used to evaluate changes in body length, using sites nested within three regions. The regions were defined with a multivariate classification tree [99, 100] that was based on measurements, using an Euclidian distance matrix with latitude as predictor. The resulting classification showed high accuracy (pseudo-r^2^ = 0.78) [99], defining the presence of three groups coincident with those detected with genetic divergence: North of 30°S, Center (one site at 31°51′S), and South (34°-42°30′S) (Additional file 16).

Principal component analysis (PCA) of morphological variables was used to evaluate geographic differences in morphology, accounting for allometric effects on body size using Thorpe’s method [101, 102]. Hotelling’s discriminant test (T^2^) was used to determine differences in shape among groups. Analyses were carried out using the software PAST [103]. We examined the allometric relationship between selected morphological variables (i.e. those contributing significantly to the PCA) and body length (Log-transformed) using an ANCOVA, with individuals pooled according to the three detected groups. Analyses were carried out using the software R [104].

PERMANOVA [48, 105] analyses were performed in order to identify significant differences between the detected groups in the PCA. The analyses were based on a Euclidian distance matrix type III (partial) and the sum of squares were calculated with 9999 permutations in the original data set.

## Additional files

Additional file 1: Raw *COI* haplotype frequencies of the 128 haplotypes of *Excirolana hirsuticauda*. (XLSX 44 kb)

Additional file 2: Pairwise population Φ_ST_ values for *COI* mtDNA of *Excirolana hirsuticauda* among 14 sites. (DOCX 79 kb)

Additional file 3: Marginal posterior probability distributions of migration rates and time since splitting between groups of *Excirolana hirsuticauda* using IMa2 with *COI* sequences. (DOCX 714 kb)

Additional file 4: Demographic reconstructions in *COI* sequences of *Excirolana hirsuticauda* using BEAST. (DOCX 183 kb)

Additional file 5: Microsatellite data of *Excirolana hirsuticauda* in GeneAlex format. (CSV 22 kb)

Additional file 6: Frequency of null alleles per locus and population for microsatellite loci of *Excirolana hirsuticauda*. (DOCX 80 kb)

Additional file 7: Locus-by-locus probability of deviation of Hardy-Weinberg Equilibrium after Bonferroni correction. (DOCX 72 kb)

Additional file 8: Summary statistics for microsatellite loci of *Excirolana hirsuticauda*. (DOCX 117 kb)

Additional file 9: *F*_*IS*_ values for microsatellite loci of *Excirolana hirsuticauda*. (DOCX 69 kb)

Additional file 10: Microsatellite pairwise population *F*_*ST*_ of *Excirolana hirsuticauda*. (DOCX 61 kb)

Additional file 11: Data of samples used for body length and morphometric analysis. (DOCX 62 kb)

Additional file 12: Body length range distribution of *Excirolana hirsuticauda*. (DOCX 137 kb)

Additional file 13: Eigenvectors of morphometric analysis. (DOCX 97 kb)

Additional file 14: Connectivity of sandy beaches across the study area based on beach length and beach separation. (DOCX 190 kb)

Additional file 15: List of the 19 morphometric measurements analysed in males of *Excirolana hirsuticauda. (DOCX 89 kb)*

Additional file 16: Multivariate classification tree based on morphological traits of *Excirolana hirsuticauda*. (DOCX 106 kb)

## Data Availability

Sequences were deposited in GenBank with accession numbers FJ532097-FJ532224. All data generated or analysed during this study are included in this published article [and its Additional files].

## Abbreviations

AMOVA: Analysis of Molecular Variance
ANCOVA: Analysis of Covariance
ANOVA: Analysis of Variance
COI: Cytochrome Oxidase I
ENSO: El Niño South Oscillation
HCS: Humboldt Current System
HKY: Hasegawa-Kishino-Yano model
HWE: Hardy-Weinberg Equilibrium
LTR: Likelihood Ratio Test
MCMC: Markov Chain Monte Carlo
Myr: Million Years
PCA: Principal Component Analysis
PERMANOVA: Permutation Analysis of Variance
PIC: Polymorphic Information Content
SAMOVA: Spatial Analysis of Molecular Variance
tMCRA: Time of the Most Recent Common Ancestor
