# Supplementary material for: Genetic and morphological divergence at a biogeographic break in the beach-dwelling brooder Excirolana hirsuticauda Menzies (Crustacea, Peracarida)
Source: BMC Evol Biol. 2019 Jun 11;19:118. doi: 10.1186/s12862-019-1442-z (PMC6560899; doi:10.1186/s12862-019-1442-z)
Supplement: Supplementary file 2 — Pairwise population ΦST values for COI mtDNA of Excirolana hirsuticauda among 14 sites. (DOCX 79 kb) [file 12862_2019_1442_MOESM2_ESM.docx]

**Genetic and morphological divergence at a biogeographic break in the beach-dwelling brooder *Excirolana hirsuticauda* Menzies (Crustacea, Peracarida).**

Pilar A. Haye, Nicolás I. Segovia, Andrea I. Varela, Rodrigo Rojas, Marcelo M. Rivadeneira & Martin Thiel

**Additional file 2**

Pairwise population Φ_ST_ values for *COI* mtDNA data among the 14 populations surveyed of *Excirolana hirsuticauda*. Significant pairwise values (*P* < 0.01) are marked in bold. Locations as in Table 1 of the main text.

|  | **CAD** | **PBL** | **COQ** | **LVI** | **MAI** | **PMU** | **PAN** | **PUR** | **TRA** | **QUE** | **CAF** | **PUÑ** | **CUC** |
| --- | --- | --- | --- | --- | --- | --- | --- | --- | --- | --- | --- | --- | --- |
| TAL | 0.013 | **0.532** | **0.558** | **0.336** | **0.311** | **0.319** | **0.352** | **0.370** | **0.342** | **0.356** | **0.509** | **0.370** | **0.319** |
| CAD |  | **0.621** | **0.674** | **0.401** | **0.374** | **0.397** | **0.418** | **0.453** | **0.408** | **0.437** | **0.605** | **0.453** | **0.384** |
| PBL |  |  | **0.586** | **0.359** | **0.333** | **0.346** | **0.375** | **0.397** | **0.365** | **0.383** | **0.536** | **0.397** | **0.342** |
| COQ |  |  |  | **0.367** | **0.340** | **0.355** | **0.384** | **0.411** | **0.374** | **0.395** | **0.567** | **0.411** | **0.349** |
| LVI |  |  |  |  | **0.065** | **0.114** | **0.173** | **0.176** | **0.169** | **0.162** | **0.324** | **0.176** | **0.146** |
| MAI |  |  |  |  |  | **0.081** | **0.145** | **0.150** | **0.144** | **0.136** | **0.298** | **0.150** | **0.121** |
| PMU |  |  |  |  |  |  | 0.004 | **0.093** | **0.075** | **0.079** | **0.142** | **0.093** | **0.061** |
| PAN |  |  |  |  |  |  |  | **0.098** | **0.077** | **0.095** | **0.080** | **0.101** | **0.075** |
| PUR |  |  |  |  |  |  |  |  | -0.008 | -0.008 | **0.345** | -0.016 | -0.006 |
| TRA |  |  |  |  |  |  |  |  |  | -0.017 | **0.290** | -0.011 | -0.003 |
| QUE |  |  |  |  |  |  |  |  |  |  | **0.330** | -0.014 | -0.011 |
| CAF |  |  |  |  |  |  |  |  |  |  |  | **0.345** | **0.282** |
| PUÑ |  |  |  |  |  |  |  |  |  |  |  |  | -0.009 |
