# Supplementary material for: Genetic and morphological divergence at a biogeographic break in the beach-dwelling brooder Excirolana hirsuticauda Menzies (Crustacea, Peracarida)
Source: BMC Evol Biol. 2019 Jun 11;19:118. doi: 10.1186/s12862-019-1442-z (PMC6560899; doi:10.1186/s12862-019-1442-z)
Supplement: Supplementary file 3 — Marginal posterior probability distributions of migration rates and time since splitting between groups of Excirolana hirsuticauda using IMa2 with COI sequences. (DOCX 714 kb) [file 12862_2019_1442_MOESM3_ESM.docx]

**Genetic and morphological divergence at a biogeographic break in the beach-dwelling brooder *Excirolana hirsuticauda* Menzies (Crustacea, Peracarida).**

Pilar A. Haye, Nicolás I. Segovia, Andrea I. Varela, Rodrigo Rojas, Marcelo M. Rivadeneira & Martin Thiel

**Additional file 3**

Marginal posterior probability frequency distribution of estimated migration rates in each direction and onset of divergence (scaled in years) of the three groups of *Excirolana hirsuticauda* (North, Center, South) estimated using *COI* sequences in Ima2. Migration probabilities between North and Center groups (a), and between the Center and South groups (b); time since the onset of divergence of North-Center groups (c), and Center-South groups (d).
