# Supplementary material for: Genetic and morphological divergence at a biogeographic break in the beach-dwelling brooder Excirolana hirsuticauda Menzies (Crustacea, Peracarida)
Source: BMC Evol Biol. 2019 Jun 11;19:118. doi: 10.1186/s12862-019-1442-z (PMC6560899; doi:10.1186/s12862-019-1442-z)

**Genetic and morphological divergence at a biogeographic break in the beach-dwelling brooder *Excirolana hirsuticauda* Menzies (Crustacea, Peracarida).**

Pilar A. Haye, Nicolás I. Segovia, Andrea I. Varela, Rodrigo Rojas, Marcelo M. Rivadeneira & Martin Thiel

**Additional file 4**

**Demographic reconstruction in *Excirolana hirsuticauda*.** Bayesian Skyline

plot mtDNA reconstructions of historical effective populations size of North and South groups estimated with *COI* sequences of *Excirolana hirsuticauda* using BEAST. The y-axis is the product of effective population size (Ne) and generation length in a log scale and the x-axis in the time in years. The median estimate is the solid white line in each group and the 95% highest posterior density (HPD) limits are in red and blue for North and South groups, respectively. Following the same color code, the thick dashed lines of each group represent the median time of the most recent common ancestor (tMRCA).


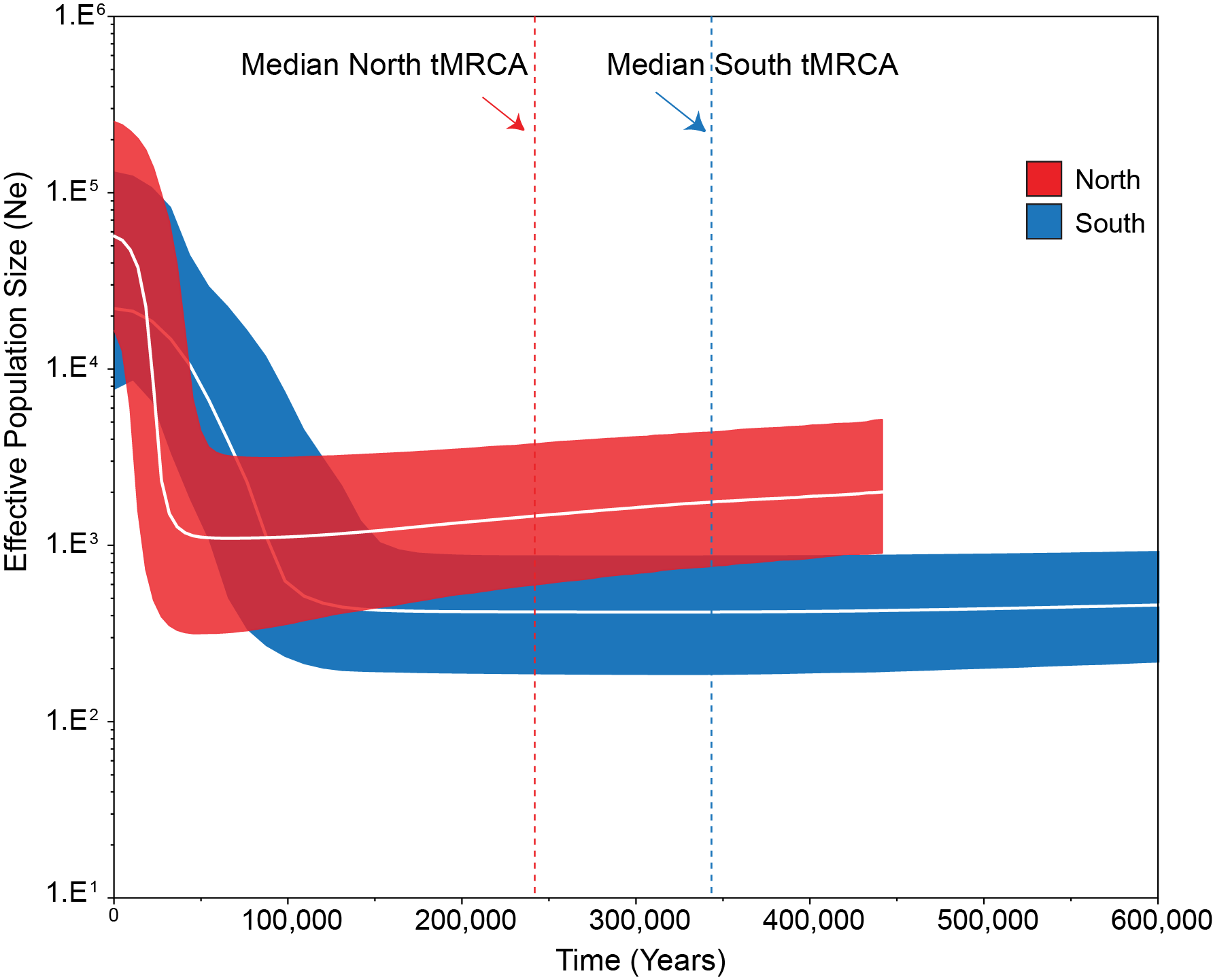

Supplement: Supplementary file 4 — Demographic reconstructions in COI sequences of Excirolana hirsuticauda using BEAST. (DOCX 183 kb) [file 12862_2019_1442_MOESM4_ESM.docx]
