# Supplementary material for: Genetic and morphological divergence at a biogeographic break in the beach-dwelling brooder Excirolana hirsuticauda Menzies (Crustacea, Peracarida)
Source: BMC Evol Biol. 2019 Jun 11;19:118. doi: 10.1186/s12862-019-1442-z (PMC6560899; doi:10.1186/s12862-019-1442-z)
Supplement: Supplementary file 6 — Frequency of null alleles per locus and population for microsatellite loci of Excirolana hirsuticauda. (DOCX 80 kb) [file 12862_2019_1442_MOESM6_ESM.docx]

**Genetic and morphological divergence at a biogeographic break in the beach-dwelling brooder *Excirolana hirsuticauda* Menzies (Crustacea, Peracarida).**

Pilar A. Haye, Nicolás I. Segovia, Andrea I. Varela, Rodrigo Rojas, Marcelo M. Rivadeneira & Martin Thiel

**Additional file 6**

Frequency of null alleles for each microsatellite locus and per local population of *Excirolana hirsuticauda* using the Brookfield method (number of initial conditions = 1000).

|  |  | ***Ehir2*** | ***Ehir4*** | ***Ehir19*** | ***Ehir38*** | ***Ehir64*** |
| --- | --- | --- | --- | --- | --- | --- |
| TAL | Brookfield | -0.0146 | 0.1285 | -0.0213 | -0.0501 | -0.0016 |
|  | Null Present | no | yes | no | no | no |
| PBL | Brookfield | 0.0038 | 0.0094 | -0.0408 | 0.0162 | -0.0786 |
|  | Null Present | no | no | no | no | no |
| COQ | Brookfield | 0.0266 | 0.0355 | 0.0695 | -0.0196 | 0.0694 |
|  | Null Present | no | no | no | no | no |
| LVI | Brookfield | -0.0864 | 0.0317 | -0.0761 | -0.0154 | -0.0162 |
|  | Null Present | no | no | no | no | no |
| PAN | Brookfield | -0.043 | -0.042 | 0.1865 | 0.0081 | 0.1508 |
|  | Null Present | no | no | yes | no | yes |
| PUR | Brookfield | 0.0465 | 0.0423 | 0.0084 | -0.0051 | 0.0962 |
|  | Null Present | no | no | no | no | no |
| QUE | Brookfield | -0.0365 | 0.0385 | 0.1763 | -0.0013 | -0.0433 |
|  | Null Present | no | no | yes | no | no |
| PUÑ | Brookfield | 0.0066 | 0.199 | -0.0521 | 0.007 | -0.0407 |
|  | Null Present | no | yes | no | no | no |
