# Supplementary material for: Genetic and morphological divergence at a biogeographic break in the beach-dwelling brooder Excirolana hirsuticauda Menzies (Crustacea, Peracarida)
Source: BMC Evol Biol. 2019 Jun 11;19:118. doi: 10.1186/s12862-019-1442-z (PMC6560899; doi:10.1186/s12862-019-1442-z)
Supplement: Supplementary file 7 — Locus-by-locus probability of deviation of Hardy-Weinberg Equilibrium after Bonferroni correction. (DOCX 72 kb) [file 12862_2019_1442_MOESM7_ESM.docx]

**Genetic and morphological divergence at a biogeographic break in the beach-dwelling brooder *Excirolana hirsuticauda* Menzies (Crustacea, Peracarida).**

Pilar A. Haye, Nicolás I. Segovia, Andrea I. Varela, Rodrigo Rojas, Marcelo M. Rivadeneira & Martin Thiel

**Additional file 7**

Microsatellite data of *Excirolana hirsuticauda*. Locus by locus probability of Hardy-Weinberg Equilibrium (HWE) and total number of loci out of HWE (Out-HWE)*.* Significant departures after Bonferroni correction in bold [*P*(HWE) < 0.05].

|  | **TAL** | **PBL** | **COQ** | **LVI** | **PAN** | **PUR** | **QUE** | **PUÑ** |
| --- | --- | --- | --- | --- | --- | --- | --- | --- |
| *Ehir2* | **0.001** | 0.642 | 0.228 | 0.82 | 0.365 | 0.057 | 0.793 | 0.535 |
| *Ehir4* | 0.124 | 0.787 | **0.004** | 0.798 | 0.841 | 0.806 | **0.011** | **0.01** |
| *Ehir19* | 0.95 | 0.802 | **0.004** | 0.108 | 0.113 | **0.01** | **0.009** | 0.811 |
| *Ehir38* | 0.949 | 0.058 | 0.435 | 0.324 | **0.08** | 0.78 | 0.187 | 0.837 |
| *Ehir64* | **0.008** | 0.142 | 0.216 | **0.003** | **0.01** | 0.169 | 0.95 | 0.95 |
| Out- HWE | 2 | 0 | 2 | 1 | 2 | 1 | 2 | 1 |
| Total | **0.193** | 0.412 | **0.079** | **0.382** | **0.248** | **0.059** | **0.63** | **0.812** |
