# Supplementary material for: Genetic and morphological divergence at a biogeographic break in the beach-dwelling brooder Excirolana hirsuticauda Menzies (Crustacea, Peracarida)
Source: BMC Evol Biol. 2019 Jun 11;19:118. doi: 10.1186/s12862-019-1442-z (PMC6560899; doi:10.1186/s12862-019-1442-z)
Supplement: Supplementary file 8 — Summary statistics for microsatellite loci of Excirolana hirsuticauda. (DOCX 117 kb) [file 12862_2019_1442_MOESM8_ESM.docx]

**Genetic and morphological divergence at a biogeographic break in the beach-dwelling brooder *Excirolana hirsuticauda* Menzies (Crustacea, Peracarida).**

Pilar A. Haye, Nicolás I. Segovia, Andrea I. Varela, Rodrigo Rojas, Marcelo M. Rivadeneira & Martin Thiel

**Additional file 8**

Summary statistics for microsatellite loci of *Excirolana hirsuticauda*. Alleles, total number of alleles; Na (Freq >= 5%), number of different alleles with a frequency >= 5%; Ne, number of effective alleles; I, Shannon's information index; AR, allelic richness after rarefaction; Ho, observed heterozygosity; He, expected heterozygosity; PIC, Polymorphic Information Content.

|  | **Locus** | ***Ehir2*** | ***Ehir4*** | ***Ehir19*** | ***Ehir38*** | ***Ehir64*** | **Total** |
| --- | --- | --- | --- | --- | --- | --- | --- |
| TAL | Alleles | 25.0 | 27.0 | 24.0 | 23.0 | 27.0 | 25.2 |
|  | Na | 3.0 | 7.0 | 2.0 | 16.0 | 5.0 | 6.6 |
|  | Ne | 2.5 | 3.0 | 1.1 | 10.5 | 2.7 | 3.9 |
|  | I | 0.973 | 1.379 | 0.173 | 2.517 | 1.211 | 1.251 |
|  | AR | 3.0 | 6.4 | 1.9 | 15.1 | 4.9 | 6.3 |
|  | Ho | 0.400 | 0.481 | 0.083 | 1.000 | 0.629 | 0.519 |
|  | He | 0.607 | 0.679 | 0.081 | 0.925 | 0.639 | 0.586 |
| PBL | Alleles | 34.0 | 35.0 | 35.0 | 35.0 | 35.0 | 34.8 |
|  | Na | 8.0 | 16.0 | 8.0 | 25.0 | 5.0 | 12.4 |
|  | Ne | 2.3 | 7.8 | 2.5 | 18.0 | 3.1 | 6.8 |
|  | I | 1.204 | 2.328 | 1.363 | 3.023 | 1.341 | 1.852 |
|  | AR | 6.4 | 12.7 | 7.1 | 20.1 | 4.9 | 10.2 |
|  | Ho | 0.529 | 0.857 | 0.657 | 0.914 | 0.771 | 0.746 |
|  | He | 0.567 | 0.885 | 0.615 | 0.958 | 0.688 | 0.743 |
| COQ | Alleles | 32.0 | 33.0 | 33.0 | 33.0 | 30.0 | 32.2 |
|  | Na | 7.0 | 9.0 | 7.0 | 21.0 | 4.0 | 11.2 |
|  | Ne | 2.5 | 4.0 | 9.4 | 14.8 | 1.8 | 6.5 |
|  | I | 1.161 | 1.623 | 2.467 | 2.839 | 0.842 | 1.786 |
|  | AR | 5.7 | 7.4 | 13.6 | 17.7 | 3.7 | 9.6 |
|  | Ho | 0.562 | 0.545 | 0.757 | 0.969 | 0.366 | 0.64 |
|  | He | 0.615 | 0.763 | 0.907 | 0.946 | 0.457 | 0.738 |
| LVI | Alleles | 33.0 | 33.0 | 27.0 | 28.0 | 31.0 | 30.4 |
|  | Na | 9.0 | 18.0 | 10.0 | 21.0 | 9.0 | 13.4 |
|  | Ne | 3.4 | 10.5 | 4.3 | 15.4 | 2.9 | 7.3 |
|  | I | 1.571 | 2.581 | 1.734 | 2.873 | 1.406 | 2.033 |
|  | AR | 7.4 | 15.2 | 8.8 | 18.7 | 7.4 | 11.5 |
|  | Ho | 0.848 | 0.848 | 0.444 | 0.964 | 0.419 | 0.705 |
|  | He | 0.724 | 0.918 | 0.779 | 0.952 | 0.667 | 0.808 |
| PAN | Alleles | 39.0 | 39.0 | 42.0 | 42.0 | 38.0 | 40.0 |
|  | Na | 9.0 | 25.0 | 15.0 | 25.0 | 10.0 | 16.8 |
|  | Ne | 4.0 | 12.4 | 7.2 | 18.1 | 3.8 | 9.1 |
|  | I | 1.663 | 2.821 | 2.221 | 3.033 | 1.675 | 2.282 |
|  | AR | 4.8 | 14.0 | 10.3 | 20.1 | 5.3 | 10.9 |
|  | Ho | 0.820 | 1.000 | 0.524 | 0.923 | 0.526 | 0.759 |
|  | He | 0.763 | 0.932 | 0.871 | 0.956 | 0.745 | 0.853 |
| PUR | Alleles | 38.0 | 38.0 | 42.0 | 42.0 | 37.0 | 39.4 |
|  | Na | 8.0 | 26.0 | 14.0 | 27.0 | 5.0 | 16.0 |
|  | Ne | 2.7 | 6.7 | 6.1 | 17.4 | 1.2 | 6.8 |
|  | I | 1.328 | 2.541 | 2.115 | 3.025 | 0.382 | 1.878 |
|  | AR | 5.6 | 16.2 | 10.3 | 17.9 | 4.0 | 10.8 |
|  | Ho | 0.579 | 0.763 | 0.500 | 0.952 | 0.108 | 0.580 |
|  | He | 0.644 | 0.862 | 0.846 | 0.954 | 0.155 | 0.692 |
| QUE | Alleles | 37.0 | 37.0 | 33.0 | 33.0 | 37.0 | 35.4 |
|  | Na | 9.0 | 24.0 | 16.0 | 20.0 | 3.0 | 14.4 |
|  | Ne | 2.5 | 5.9 | 8.3 | 16.0 | 1.2 | 6.8 |
|  | I | 1.323 | 2.494 | 2.361 | 2.855 | 0.352 | 1.877 |
|  | AR | 6.9 | 17.2 | 12.9 | 17.5 | 2.5 | 11.4 |
|  | Ho | 0.648 | 0.648 | 0.606 | 0.939 | 0.189 | 0.606 |
|  | He | 0.611 | 0.844 | 0.892 | 0.952 | 0.175 | 0.695 |
| PUÑ | Alleles | 31.0 | 27.0 | 35.0 | 35.0 | 32.0 | 32.0 |
|  | Na | 8.0 | 13.0 | 8.0 | 23.0 | 2.0 | 10.8 |
|  | Ne | 2.9 | 3.4 | 1.7 | 13.8 | 1.2 | 4.6 |
|  | I | 1.376 | 1.792 | 0.949 | 2.863 | 0.274 | 1.451 |
|  | AR | 6.5 | 11.3 | 6.1 | 18.5 | 2.0 | 8.9 |
|  | Ho | 0.645 | 0.407 | 0.457 | 0.914 | 0.156 | 0.516 |
|  | He | 0.671 | 0.716 | 0.418 | 0.941 | 0.146 | 0.578 |
| Average AR | | 5.8 | 12.7 | 8.9 | 18.2 | 4.4 |  |
| Total HO | | 0.687 | 0.782 | 0.438 | 0.855 | 0.326 | 0.634 |
| Total HE | | 0.693 | 0.923 | 0.572 | 0.862 | 0.467 | 0.721 |
