# Supplementary material for: Genetic and morphological divergence at a biogeographic break in the beach-dwelling brooder Excirolana hirsuticauda Menzies (Crustacea, Peracarida)
Source: BMC Evol Biol. 2019 Jun 11;19:118. doi: 10.1186/s12862-019-1442-z (PMC6560899; doi:10.1186/s12862-019-1442-z)
Supplement: Supplementary file 9 — FIS values for microsatellite loci of Excirolana hirsuticauda. (DOCX 69 kb) [file 12862_2019_1442_MOESM9_ESM.docx]

**Genetic and morphological divergence at a biogeographic break in the beach-dwelling brooder *Excirolana hirsuticauda* Menzies (Crustacea, Peracarida).**

Pilar A. Haye, Nicolás I. Segovia, Andrea I. Varela, Rodrigo Rojas, Marcelo M. Rivadeneira & Martin Thiel

**Additional file 9**

Microsatellite loci of *Excirolana hirsuticauda*. Locus-by-locus and per population *F_IS_* values*.* Significant values are in bold (*P* < 0.05).

|  | **TAL** | **PBL** | **COQ** | **LVI** | **PAN** | **PUR** | **QUE** | **PUÑ** |
| --- | --- | --- | --- | --- | --- | --- | --- | --- |
| *Ehir2* | **0.346** | 0.068 | 0.087 | -0.174 | -0.077 | 0.102 | -0.062 | 0.039 |
| *Ehir4* | **0.295** | 0.032 | **0.288** | 0.077 | -0.075 | **0.117** | **0.234** | **0.436** |
| *Ehir19* | -0.022 | -0.069 | **0.168** | **0.434** | **0.401** | **0.412** | **0.324** | -0.096 |
| *Ehir38* | -0.084 | 0.046 | -0.025 | -0.013 | 0.029 | 0.002 | 0.013 | 0.029 |
| *Ehir64* | 0.016 | -0.123 | 0.202 | **0.376** | **0.297** | **0.308** | -0.077 | -0.069 |
| Total | **0.117** | -0.004 | **0.134** | **0.130** | **0.111** | **0.163** | **0.129** | **0.110** |
