# Supplementary material for: Genetic and morphological divergence at a biogeographic break in the beach-dwelling brooder Excirolana hirsuticauda Menzies (Crustacea, Peracarida)
Source: BMC Evol Biol. 2019 Jun 11;19:118. doi: 10.1186/s12862-019-1442-z (PMC6560899; doi:10.1186/s12862-019-1442-z)
Supplement: Supplementary file 10 — Microsatellite pairwise population FST of Excirolana hirsuticauda. (DOCX 61 kb) [file 12862_2019_1442_MOESM10_ESM.docx]

**Genetic and morphological divergence at a biogeographic break in the beach-dwelling brooder *Excirolana hirsuticauda* Menzies (Crustacea, Peracarida).**

Pilar A. Haye, Nicolás I. Segovia, Andrea I. Varela, Rodrigo Rojas, Marcelo M. Rivadeneira & Martin Thiel

**Additional file 10**

Pairwise multi-locus *F_ST_* between 8 sampled local populations of *Excirolana hirsuticauda* using data from microsatellite loci. Significant values in bold (*P* < 0.05).

|  | TAL | PBL | COQ | LVI | PAN | PUR | QUE | PUÑ | Total |
| --- | --- | --- | --- | --- | --- | --- | --- | --- | --- |
| TAL | **-** | **0.156** | **0.187** | **0.193** | **0.206** | **0.302** | **0.322** | **0.391** | **0.251** |
| PBL |  | **-** | 0.069 | **0.149** | **0.134** | **0.23** | **0.236** | **0.306** | **0.183** |
| COQ |  |  | - | **0.135** | **0.145** | **0.238** | **0.24** | **0.295** | **0.187** |
| LVI |  |  |  | - | **0.027** | **0.144** | **0.152** | **0.236** | **0.148** |
| PAN |  |  |  |  | - | 0.074 | **0.082** | **0.169** | **0.16** |
| PUR |  |  |  |  |  | - | 0 | **0.1** | **0.227** |
| QUE |  |  |  |  |  |  | - | **0.089** | **0.155** |
| PUÑ |  |  |  |  |  |  |  | - | **0.121** |
| Total |  |  |  |  |  |  |  |  | **0.179** |
