# Supplementary material for: Genetic and morphological divergence at a biogeographic break in the beach-dwelling brooder Excirolana hirsuticauda Menzies (Crustacea, Peracarida)
Source: BMC Evol Biol. 2019 Jun 11;19:118. doi: 10.1186/s12862-019-1442-z (PMC6560899; doi:10.1186/s12862-019-1442-z)
Supplement: Supplementary file 11 — Data of samples used for body length and morphometric analysis. (DOCX 62 kb) [file 12862_2019_1442_MOESM11_ESM.docx]

**Genetic and morphological divergence at a biogeographic break in the beach-dwelling brooder *Excirolana hirsuticauda* Menzies (Crustacea, Peracarida).**

Pilar A. Haye, Nicolás I. Segovia, Andrea I. Varela, Rodrigo Rojas, Marcelo M. Rivadeneira & Martin Thiel

**Additional file 11**

Localities, acronyms, latitude and number of individuals of *Excirolana hirsuticauda* analysed per site for body length measurements and morphometric analyses. N, sample size.

| **Location** | **Acronym** | **Latitude** | **N Body size** | **N Morphometry** |
| --- | --- | --- | --- | --- |
| Caldera | CAD | 26º59’S | 399 | 10 |
| Punta Choros | PCH | 29º15’S | 455 | 10 |
| Coquimbo | COQ | 29º54’S | 200 | 20 |
| Los Vilos | LVI | 31º51’S | 589 | 20 |
| Pichilemu | PMU | 34º23’S | 501 | 10 |
| Calfuco | CAF | 39º46’S | 636 | 10 |
| Cucao | CUC | 42º40’S | 582 | 10 |
| **TOTAL** |  |  | **3,362** | **90** |
