# Supplementary material for: Genetic and morphological divergence at a biogeographic break in the beach-dwelling brooder Excirolana hirsuticauda Menzies (Crustacea, Peracarida)
Source: BMC Evol Biol. 2019 Jun 11;19:118. doi: 10.1186/s12862-019-1442-z (PMC6560899; doi:10.1186/s12862-019-1442-z)
Supplement: Supplementary file 13 — Eigenvectors of morphometric analysis. (DOCX 97 kb) [file 12862_2019_1442_MOESM13_ESM.docx]

**Genetic and morphological divergence at a biogeographic break in the beach-dwelling brooder *Excirolana hirsuticauda* Menzies (Crustacea, Peracarida).**

Pilar A. Haye, Nicolás I. Segovia, Andrea I. Varela, Rodrigo Rojas, Marcelo M. Rivadeneira & Martin Thiel

**Additional file 13**

Values of the eigenvectors (variables-morphometric measurements) from morphometric analyses of *Excirolana hirsuticauda*, indicating the contribution of each morphometric measurement to the principal components. Table shows the first three Principal Components of each of the 19 considered variables. Details of variables in Additional file 15. Significant values marked in bold.

| **Variable** | **PC1** | **PC2** | **PC3** |
| --- | --- | --- | --- |
| Greatest body width | -0.001 | -0.016 | 0.153 |
| Interocular distance | 0.039 | -0.046 | 0.211 |
| Pleotelson length | 0.042 | -0.034 | 0.268 |
| Setae number on posterior margin of pleotelson | -0.223 | -0.383 | 0.14 |
| Maximum length of setae from posterior margin of pleotelson | -0.135 | -0.078 | 0.132 |
| Uropodal endopod length | 0.012 | -0.061 | 0.209 |
| Total length of antenna 1 | 0.055 | 0.011 | 0.279 |
| Total length of antenna 2 | 0.008 | -0.084 | 0.314 |
| Length of peduncle A1 | -0.028 | -0.085 | 0.204 |
| Length of flagellum A1 | 0.089 | 0.052 | 0.312 |
| Length of peduncle A2 | -0.004 | -0.07 | 0.218 |
| Length of flagellum A2 | 0.015 | -0.095 | 0.387 |
| Length of the 4th peduncular article A2 | 0.035 | -0.098 | 0.184 |
| Article number of flagellum A1 | -0.257 | -0.27 | -0.002 |
| Article number of flagellum A2 | **-0.535** | **-0.566** | -0.177 |
| *Appendix masculina* length | -0.072 | -0.068 | 0.144 |
| Length of endopod on pleopod 2 | 0.08 | -0.016 | 0.281 |
| Length of the lateral projection of the *appendix masculina* | **-0.742** | **0.633** | 0.181 |
| Length of pereopod 3 | 0.014 | -0.033 | 0.262 |
