# Supplementary material for: Genetic and morphological divergence at a biogeographic break in the beach-dwelling brooder Excirolana hirsuticauda Menzies (Crustacea, Peracarida)
Source: BMC Evol Biol. 2019 Jun 11;19:118. doi: 10.1186/s12862-019-1442-z (PMC6560899; doi:10.1186/s12862-019-1442-z)
Supplement: Supplementary file 14 — Connectivity of sandy beaches across the study area based on beach length and beach separation. (DOCX 190 kb) [file 12862_2019_1442_MOESM14_ESM.docx]

**Genetic and morphological divergence at a biogeographic break in the beach-dwelling brooder *Excirolana hirsuticauda* Menzies (Crustacea, Peracarida).**

Pilar A. Haye, Nicolás I. Segovia, Andrea I. Varela, Rodrigo Rojas, Marcelo M. Rivadeneira & Martin Thiel

**Additional file 14**

Connectivity of sandy beaches across the study area based on data beach length and beach separation along the study area obtained from Thiel et al. (2007). Connectivity was measured using the expression $C_{i}= \sum\left( e^{d_{ij}}*L_{j} \right)$, where $C_{i}$ is the connectivity of the $i$-esim beach, the $d_{ij}$is the minimum (edge) distance between beaches, and $L_{j}$ is length of the $j$ beach (for $i\neq j)$. Sandy beaches were mapped using Google Earth ®.

­­
