# Supplementary material for: Genetic and morphological divergence at a biogeographic break in the beach-dwelling brooder Excirolana hirsuticauda Menzies (Crustacea, Peracarida)
Source: BMC Evol Biol. 2019 Jun 11;19:118. doi: 10.1186/s12862-019-1442-z (PMC6560899; doi:10.1186/s12862-019-1442-z)
Supplement: Supplementary file 15 — List of the 19 morphometric measurements analysed in males of Excirolana hirsuticauda. (DOCX 89 kb) [file 12862_2019_1442_MOESM15_ESM.docx]

**Genetic and morphological divergence at a biogeographic break in the beach-dwelling brooder *Excirolana hirsuticauda* Menzies (Crustacea, Peracarida).**

Pilar A. Haye, Nicolás I. Segovia, Andrea I. Varela, Rodrigo Rojas, Marcelo M. Rivadeneira & Martin Thiel

**Additional file 15**

List of morphometric measurements analysed in males of *Excirolana hirsuticauda*.

|  | Measurements |
| --- | --- |
| 1 | Largest body width: measured at 5th pereonal segment. |
| 2 | Interocular distance: distance between eyes. |
| 3 | Pleotelson length: anterior margin to the posterior end. |
| 4 | Setae number on posterior margin of pleotelson |
| 5 | Maximum length of setae from posterior margin of pleotelson: measured in the largest seta. |
| 6 | Uropodal endopod length: proximal margin to the tip of the endopod. |
| 7 | Total length of antenna 1: proximal margin of peduncle to the tip of flagellum. |
| 8 | Total length of antenna 2: as for A1. |
| 9 | Length of peduncle A1: proximal to distal margin of peduncle. |
| 10 | Length of flagellum A1: proximal margin of first article to tip of flagellum |
| 11 | Length of peduncle A2 |
| 12 | Length of flagellum A2 |
| 13 | Length of the 4th peduncular article A2 |
| 14 | Article number of flagellum A1 |
| 15 | Article number of flagellum A2 |
| 16 | Appendix masculina length: base to tip of the appendix. |
| 17 | Length of endopod on pleopod 2: base to the tip. |
| 18 | Length of the lateral projection of the appendix masculina: base to the tip of the projection. |
| 19 | Length of pereopod 3: proximal margin of the basis to the tip of the dactylus. |
