# Supplementary material for: Genetic and morphological divergence at a biogeographic break in the beach-dwelling brooder Excirolana hirsuticauda Menzies (Crustacea, Peracarida)
Source: BMC Evol Biol. 2019 Jun 11;19:118. doi: 10.1186/s12862-019-1442-z (PMC6560899; doi:10.1186/s12862-019-1442-z)
Supplement: Supplementary file 16 — Multivariate classification tree based on morphological traits of Excirolana hirsuticauda. (DOCX 106 kb) [file 12862_2019_1442_MOESM16_ESM.docx]

**Genetic and morphological divergence at a biogeographic break in the beach-dwelling brooder *Excirolana hirsuticauda* Menzies (Crustacea, Peracarida).**

Pilar A. Haye, Nicolás I. Segovia, Andrea I. Varela, Rodrigo Rojas, Marcelo M. Rivadeneira & Martin Thiel

**Additional file 16**

Multivariate classification tree based on all morphological traits measured for *Excirolana hirsuticauda* (list of measurements in Additional file 15). The analysis reveals 3 clusters Analyses were carried out using the package ‘mvpart’ (De’ath, 2014) in R.
